# Lead Isotopes Combined with Geochemical Baseline in Sediments: A Novel Tool to Trace Anthropogenic Pb Sources

**DOI:** 10.3390/ijerph17031112

**Published:** 2020-02-10

**Authors:** Dongyu Xu, Bo Gao

**Affiliations:** 1 State Key Laboratory of Simulation and Regulation of Water Cycle in River Basin, China Institute of Water Resources and Hydropower Research, Beijing 100038, China; xudy@iwhr.com; 2 Department of Water Environment, China Institute of Water Resources and Hydropower Research, Beijing 100038, China

**Keywords:** lead (Pb), regional geochemical baseline, Pb isotopes, sediments, source identification

## Abstract

Traditional Pb isotopic identification only based on total Pb concentration and Pb isotopic ratios, resulted in difficulty for tracing Pb sources in the complex environmental medium, especially for sediment. Herein, a novel approach combining with regional geochemical baseline (RGB) and Pb isotopic ratios are used to directly trace anthropogenic Pb sources and calculate Pb source appointments in sediment. In this study, total Pb concentrations and isotopic ratios were analyzed for a 7-m long sediment core (92 sediment samples) collected from a reservoir. RGB of Pb was used to calculate anthropogenic Pb concentrations (R_d_), their contributions (C_Rd_) and screen the sediments influenced by anthropogenic activities. Among those sediments influenced by anthropogenic activities, a positive correlation was found between ^206^Pb/^207^Pb ratios and R_d_, indicating there were two anthropogenic Pb sources in sediment. Further source identification using ^206^Pb/^207^Pb and ^208^Pb/^207^Pb indicated that these two anthropogenic Pb sources originated from coal consumption and aerosol input. Finally, C_Rd_ and Pb isotopic ratios were used to calculate these two Pb source appointments (1.13% for coal consumption and 7.53% for aerosol input). This study demonstrated that source identification using RGB and Pb isotopes could be a novel attempt for identifying anthropogenic Pb sources in sediment.

## 1. Introduction

Trace metals in aquatic environments are a source of substantial concern due to their toxicity, persistence, and biological enrichment by the food chain [1,2]. In fact, sediments are a reservoir of trace metal contaminants. Over 90% of the metals in aquatic systems occur in the sediments or suspended particles [3,4]. In the aquatic environment, various physicochemical processes (e.g., precipitation, adsorption, and chelation) lead to the deposition of trace metals in sediments [5]. On the other hand, due to the change of the physicochemical conditions (e.g., pH, Eh, sediment resuspension, desorption, and redox reaction), sediment is also a potential source of trace metals as it has the potential to rerelease the trace metals back into the water column [6]. Therefore, sediment is the sink and source for trace metals in aquatic ecosystems. It is also an important medium for understanding the geochemical characteristics and environmental behavior of trace metals. 

The effective measurement of protecting water quality is to control and reduce the anthropogenic input of pollutants. Source identification of pollutants is a key scientific issue that cannot be ignored. As a toxic metal, lead (Pb) contamination in sediments has been reported worldwide due to the wide industrial uses and serious threat to human health, particularly when bioaccumulated in the tissues of organisms and biomagnified through the food web [7]. Pb in the environment is generally released by the natural weathering of parent rocks and anthropogenic sources (e.g., gasoline, coal combustion, mining activities, and industrial emissions) [8]. In fact, strong efforts to identify metal pollution sources entering the aquatic ecosystems have been attempted in the past several decades (e.g., principal component analysis, clustering analysis, and multivariate statistical analysis) [9,10,11]. These traditional methods usually require large databases and sophisticated statistics and cannot be used to accurately identify the potential origins of trace metals [12]. Pb’s stable isotope is one of the most powerful isotopic fingerprinting tools to differentiate the various sources of Pb among complex environmental matrices [12]. Nevertheless, due to the high complexity and heterogeneity of the sediments, it was difficult to trace Pb sources only by establishing the relationship between the total Pb concentrations and their corresponding Pb isotopic ratios [12,13]. Based on these reasons, a new development in the application of Pb isotopes is needed to coordinate with other information, including the geographic information system (GIS) mapping, other elements’ isotopes, and so on [14,15,16]. In recent years, the regional geochemical baseline (RGB) of trace metals has been recognized as a useful tool to distinguish between trace metals from anthropogenic input and natural sources [17,18]. In fact, the essence of the source identification of Pb pollution is to identify the anthropogenic Pb sources. Assuming that there was a direct relationship between anthropogenic input information and these corresponding Pb isotopic ratios, this would provide useful information to assist the identification of Pb sources in sediments. In this context, the major objectives of this study were to (1) establish the RGB model and calculate the amount and contribution of anthropogenic Pb in sediments; (2) establish the relationship between the amount of anthropogenic Pb and their corresponding Pb isotopic ratios; and (3) determine the anthropogenic Pb sources and their contributions by combining the RGB model and Pb isotopic ratios. To the best of our knowledge, our effort is a good attempt and enriches the method of Pb isotopic identification with the help of the geochemical approach in sediments.

## 2. Materials and Methods

### 2.1. Sampling Sites

Daheiting Reservoir (DHTR) is located in the junction between the cities of Chengde and Tangshan in Hebei province. The function of this reservoir is to carry the water from its upper reservoir (the Panjiakou reservoir) and then raise the water level to provide water for both Tianjin and Tangshan. The total capacity of DHTR is 337 million m^3^. A 7-m long sediment core was obtained in downstream of DHTR. The first 1 m of this sediment core was cut into 2 cm long sections, and the other sediment core was cut into 5 cm long sections. The sediment samples were placed in clean polyethylene bags and treated immediately upon returning to the laboratory. All samples were freeze-dried, gently crushed and ground in an agate mortar, and passed through a 0.25 mm nylon sieve for further analysis.

### 2.2. Measurement of Trace Metals Concentrations

All chemical treatments were performed in an ultraclean laboratory and all reagents were of a high purity grade. A strong acid digestion method (HNO_3_ + H_2_O_2_ + HF) was used to digest the samples in solution [19]. The digested solutions were measured using Inductively Coupled Plasma-mass Spectrometry (ICP-MS, Perkin Elmer Elan DRC-e) for Pb and Li concentrations. Quality control of trace metals was tested by certified reference materials of the stream sediment (GSD-1a), which were purchased from the Chinese Institute of Geophysical and Geochemical Exploration. Analytical results agreed well with the certified values (the recovery rates of Pb and Li were 102.35% and 96.31%, respectively). 

### 2.3. Analysis of Pb Isotopic Composition 

Isotopes of Pb were separated for analyses using ion-exchange microcolumns of Dowex-I anion resin (200–400 mesh) and HBr and HCl as eluents [20]. Measurements of Pb isotopic composition were carried out using an ICP-MS (Perkin Elmer Elan DRC-e). The average measured values of the standard NIST SRM-981 were ^206^Pb/^207^Pb = 1.094 ± 0.001 and ^207^Pb/^208^Pb = 0.422 ± 0.002 (N = 20), respectively, which were in close agreement with the certified standard values (1.093 and 0.422, respectively). The relative standard deviations (RSD) of Pb isotopic ratios were generally <0.5% (N = 20). 

### 2.4. Statistical Analysis

Statistical analyses were carried out using the SPSS 17.0 software and the Sigmaplot 10.0 for Windows. Pearson’s correlation was used to analyze the significance level in this study. 

## 3. Results and Discussion

### 3.1. Tracing Pb Sources Using the Traditional Pb Isotopic Approach

**Figure 1:**
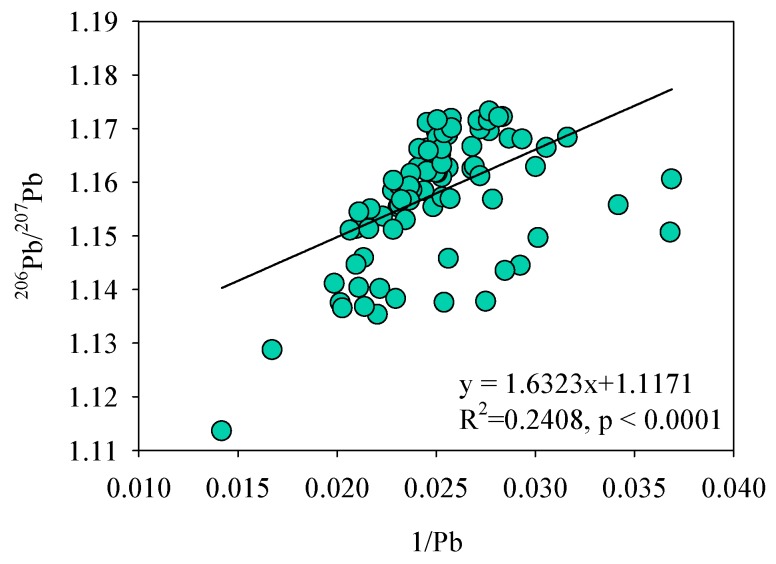
Relationships between Pb contents and ^206^Pb/^207^Pb ratios before screening by regional geochemical baseline (RGB).

Isotopic identification has been successfully applied for tracing Pb sources in different environmental matrices. In terms of Pb, the traditional method investigated the relationship between ^206^Pb/^207^Pb ratios and the reciprocal of Pb concentrations/Pb concentrations to establish a linear equation to identify Pb sources [21]. The simple binary model was commonly used to calculate the approximate contributions of the two end-members (i.e., background Pb vs. anthropogenic Pb input) [22]. In this study, there was a weak correlation between the ^206^Pb/^207^Pb ratios and the reciprocal of Pb concentrations in sediments (R^2^ = 0.2408) (Figure 1), implying that more than two possible sources of Pb in this sediment profile [23]. In this condition, the Pb sources cannot be identified easily because sediments are heterogeneous and it is difficult to ascertain the Pb sources in sediment core [13]. Previous study suggests that other identification methods are needed to help in tracing Pb sources (e.g., GIS or isotopic information of other elements) [12]. However, these methods need extensive calculations, experiments, and complicated isotopic instruments. Therefore, it is necessary to find assistant methods to solve the problem during the process of tracing Pb sources in aquatic environments. 

### 3.2. Establishment of Pb Geochemical Baseline in Sediment Core

**Figure 2:**
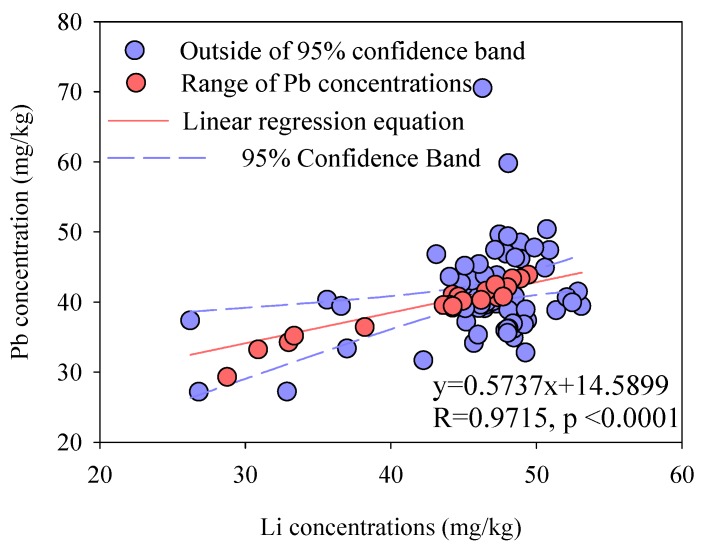
The process of establishing the RBG-Pb model.

The RGB model was regarded as an effective tool to differentiate between anthropogenic and natural sources in soils or sediments [17,18,24]. In this study, the RGB model was used to distinguish between anthropogenic and natural Pb sources in 7-m long sediment core. The RGB of Pb (RGB-Pb) in the sediment core and the corresponding RGB-Pb model were calculated by the normalization method and are presented in Figure 2 [25]. 

Lithium (Li) was chosen as the inert element to calculate the RGB-Pb in this study because Li could avoid the influence of the anthropogenic inputs and the interference of particle size in sediments [17,25]. Firstly, the linear regression equation between Pb and Li should be established as followed:

C_Pb_ = a × C_Li_ + b (1)

where C_Pb_ and C_Li_ are the concentrations of Pb and Li in the sediment core, respectively, mg/kg; a and b are the regression constants. In the x-y scatterplot described by Equation (1), according to the method reported previously [25], the data outside the 95% confidence limit were characterized as anthropogenic sources and were removed. The remaining data inside the 95% confidence limit were characterized as natural sources. These remaining data were re-established as a new regression equation, then obtained new regression constants (c and d) were established as the RGB-Pb model following the equation:

(2) $$B_{m}=c\times \bar{C_{Li}}+d$$

where B_m_ is the RGB-Pb, mg/kg; $\bar{C_{Li}}$ is the average concentration of Li samples inside the 95% confidence limit, mg/kg; c and d are the new regression constants. Using the average concentration of remaining data for Li, the naturally sourced Pb concentration was obtained. This Pb value was defined as the value of RGB-Pb. In terms of above Equation (2), the B_m_ (RGB-Pb) was 39.63 mg/kg, which was approximate to the average of Pb in sediment core (40.77 mg/kg), implying that there was only a slight accumulation of Pb in the sediment core.

### 3.3. Distinguish Anthropogenic and Natural Pb Sources Using RGB-Pb

**Figure 3:**
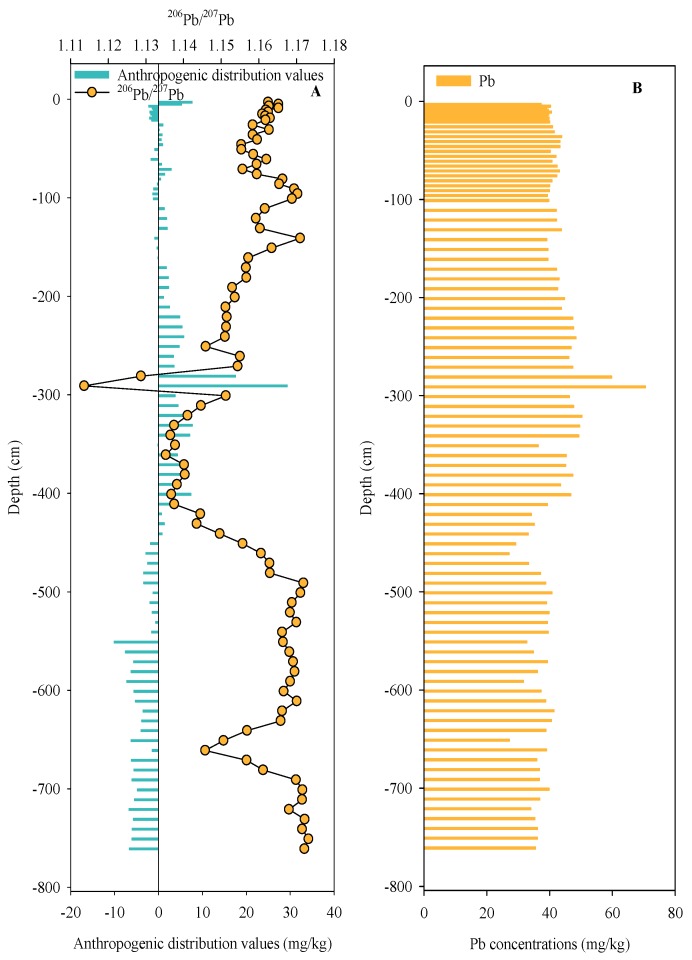
Pb concentrations, distribution characteristics of anthropogenic distribution values, and ^206^Pb/^207^Pb in sediment profile ((**A**) presents the distribution values of R_d_ and ^206^Pb/^207^Pb in sediment profile; (**B**) presents the distribution of Pb concentrations in sediment profile).

According to the RGB-Pb model, the RGB-Pb in each sample (B_mi_, i = 92) could be calculated [18]. Then, in terms of these B_mi_ values, the anthropogenic Pb concentrations (R_d_) were the values of the real Pb concentrations (D_mi_) minus their corresponding B_mi_ in each sample (Equation (3)) (Figure 3 A,B). Using the R_d_ values could differentiate between natural sources and anthropogenic sources (R_d_ > 0 indicating anthropogenic input; R_d_ ≤ 0 indicating natural input). Furthermore, the percentage of anthropogenic contribution (C_Rd_) in the sediment core could be calculated by Equation (4).

R_d_ = D_mi_ − B_mi_, i = 1…92 (3)

C_Rd_ (%) = 100 × R_d_/B_mi_ (4)

**Figure 4:**
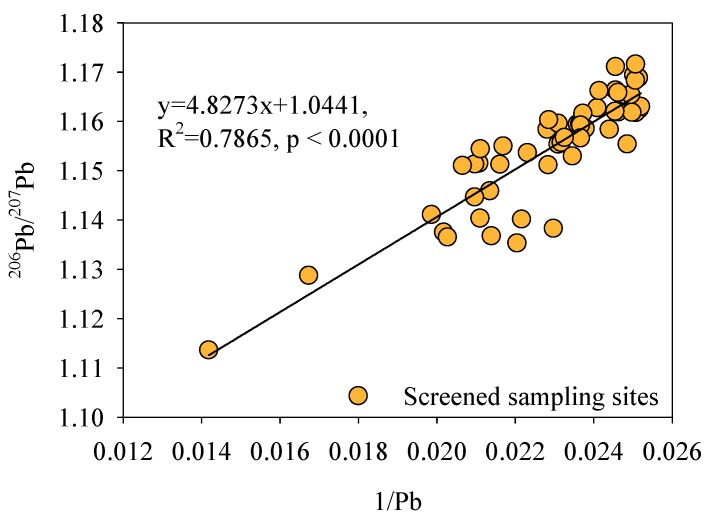
Relationships between Pb contents and ^206^Pb/^207^Pb ratios after screening by RGB.

According to Equation (4), the average of the C_Rd_ (anthropogenic contribution) value was 8.66%, which also suggested that the Pb slightly accumulated in the sediment core and mainly originated from a natural source. In addition, according to Figure 3A, most R_d_ exhibited positive values from the depth of −2 m to −4 m in the sediment core, indicating that anthropogenic input was present in these samples. To determine the sources of the anthropogenic Pb input in these samples, the two parameters (R_d_ and the Pb isotope) in these samples were used for further analysis of the Pb sources. Interestingly, there was an opposite trend between the R_d_ and the distribution characteristics of the ^206^Pb/^207^Pb ratios in these positive samples (Figure 3A). Moreover, the correlation analysis showed that a negative correlation truly existed between the R_d_ (R_d_ > 0) and ^206^Pb/^207^Pb ratios (R^2^ = 0.7865, *p* < 0.0001) (Figure 4), indicating that the binary model can identify the Pb sources in these selected anthropogenic sediments after screening by RGB model. Hence, our results demonstrated that R_d_ and Pb isotopic ratios were effective parameters for tracing anthropogenic Pb in sediments compared with the traditional isotopic identification method that used the reciprocal of Pb concentrations/Pb concentrations and Pb isotopic ratios. 

### 3.4. Source Identification of Anthropogenic Pb in Sediments

**Figure 5:**
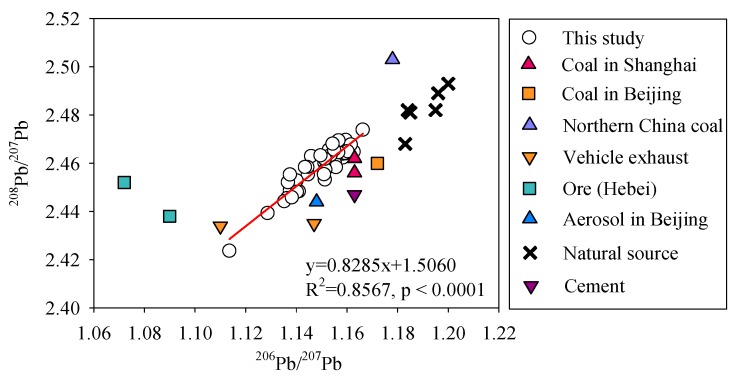
Isotopic composition (^208^Pb/^207^Pb versus ^206^Pb/^207^Pb) in sediment.

To further identify the sources of anthropogenic Pb deposited in the sediments, the measured isotope ratios were compared to those of source-specific materials. The Pb isotopic compositions of those source-specific materials are listed in Table 1. Overall, the average value of the ^206^Pb/^207^Pb ratios and ^208^Pb/^207^Pb for different anthropogenic Pb samples were 1.150 and 2.458, respectively. The Pb isotopic ratios in anthropogenic Pb samples screened by RGB model located nearly to the samples of coal and aerosol, indicating anthropogenic Pb should be homologous with the values of coal and aerosol (Figure 4). This result meant that coal consumption and aerosol deposition might be the sources of the anthropogenic Pb in sediments. Additionally, the linear correlation of the Pb isotope ratios (^206^Pb/^207^Pb vs. ^208^Pb/^207^Pb) in these samples was significant (R^2^ = 0.8567) (Figure 5), also suggesting that the Pb in the sediments fit a binary mixing model. This model indicated that sediments could receive Pb from two sources (Figure 5). Consequently, the anthropogenic Pb originated from the coal and the aerosol in this study. Moreover, this result further suggests that the atmospheric deposition of coal combustion dust might be speculated to be the major transportation pathway for anthropogenic Pb input to DHTR sediments.

To quantify the Pb contributions of coal consumption and aerosol input (anthropogenic sources) in sediments, the following binary mixing model was adopted [36]:

*R_anthropogenic_* = *R_coal_ X_coal_* + *R_aerosol_ X_aerosol_* (5)

*X_coal_* + *X_aerosol_* = 1 (6)

where *R_anthropogenic_* is the average ^206^Pb/^207^Pb ratio of the anthropogenic Pb calculated from the screened samples by RGB model with a value of 1.150. The *R_coal_* and *R_aerosol_* referred to the previous data of the ^206^Pb/^207^Pb ratios (1.163 for *R_coal_* and 1.148 for *R_aerosol_*) [27,28]. According to Equations (5) and (6), the contributions of all anthropogenic Pb sources were calculated. Because the anthropogenic contribution of Pb was 8.66%, the results reveal that the anthropogenic source contributions were 1.13% for coal consumption and 7.53% for aerosol input, respectively. These anthropogenic percentage values also demonstrated that the atmospheric deposition of coal combustion dust was the main transportation pathway of Pb in the study area. The result also correlated with a high incidence of fog and haze in North China. A previous study also observed that Pb was the most abundant element, independent of the particulate matter (PM) pollution levels during the wintertime in Beijing [37]. With the help of the RGB-Pb model, this study provided a novel approach to accurately quantify the contributions of multiple anthropogenic Pb sources using Pb isotopic ratios in sediments. 

## 4. Conclusions 

In summary, considering the essence of the identification of sources, the amount of anthropogenic Pb (R_d_) was calculated by the RGB model, and a relationship was established between R_d_ and Pb isotopic ratios. This method has broken through the traditional isotopic identification method using the correlation analysis between Pb concentrations/the reciprocal of Pb concentrations and Pb isotopic ratios. In addition, the combination of the anthropogenic Pb contribution by the RGB model with Pb isotopic ratios could accurately identify the different Pb sources in sediments. Moreover, compared with the traditional method, there were two advantages, as follows: (1) the novel approach directly identified the anthropogenic sources, which made it possible to identify the source of complex sediments. (2) This method also simplified the process of Pb source identification and reduced the workload of source identification. This combination approach will make it possible to trace pollution sources of other elements in the future.

## Figures and Tables

**Table 1:** Values of Pb isotope ratio of different sources.

	^206^Pb/^207^Pb	^208^Pb/^207^Pb	References
Anthropogenic Pb in sediments	1.150	2.458	This study
Aerosol in Beijing	1.148	2.444	[26]
Coal in Shanghai	1.163	2.462	[27]
Coal in Shanghai	1.163	2.456	[27]
Cement	1.163	2.447	[27]
Coal in Beijing	1.172	2.460	[28]
Northern China coal	1.178	2.503	[29]
Vehicle exhaust (leaded)	1.110	2.434	[30]
Vehicle exhaust(unleaded)	1.147	2.435	[30]
Ore (Hebei)	1.072	2.452	[31]
Ore (Hebei)	1.090	2.438	[31]
Natural source	1.184	2.482	[32]
	1.183	2.468	[32]
	1.195	2.482	[33]
	1.196	2.489	[34]
	1.185	2.481	[34]
	1.200	2.493	[35]
